# Tap Water and Trihalomethanes: Flow of Concerns Continues

**Published:** 2005-07

**Authors:** Ernie Hood

Trihalomethanes (THMs) are the result of a reaction between the chlorine used for disinfecting tap water and natural organic matter in the water. At elevated levels, THMs have been associated with negative health effects such as cancer and adverse reproductive outcomes. Now a study by government and academic researchers adds to previous evidence that dermal absorption and inhalation of THMs associated with everyday tap water use can result in significantly higher blood THM concentrations than simply drinking the water does **[*EHP* 113:863–870]**. The results of this exposure assessment study could serve as a guide for future epidemiologic investigations exploring the potential connection between THMs in tap water and adverse health effects.

The researchers recruited seven healthy participants aged 21–30 years to spend two 24-hour periods (usually one week apart) in one of two study residences. The study residences were typical ranch-style homes, one located in North Carolina, the other in Texas. The North Carolina house was served with a water supply higher in THMs than that of the Texas house.

Over the total two days, each participant performed 14 activities using tap water. These included drinking a hot beverage prepared with tap water (THM-free bottled water was consumed except when drinking was part of a test activity), washing their hands, showering, washing dishes both by hand and in a dishwasher, and washing clothes in a washing machine. The water use activities were rigidly scheduled and controlled for exposure time and water temperature.

The team took baseline measurements of the THMs in ambient indoor air, cold tap water, and subjects’ blood and exhaled breath just before and just after each activity. The ratio between pre- and post-activity measurements illustrated the impact of each activity on participants’ blood and exhaled breath THM concentrations; twofold or greater deviation from baseline was established as meaningful.

Relatively high pre- to postactivity ratios were observed for several of the activities. For example, blood concentrations rose 5- to 15-fold as a result of showering in the North Carolina participants, and rose approximately 5-fold in the Texas subjects. The results confirm that showering and bathing are important sources of THM exposure; they also provide evidence that other THM exposure scenarios, such as washing dishes by hand and being exposed to a cohabitant’s shower steam, may also be important.

Although an apparent dose–response relationship was discovered, the authors emphasize that public health implications should not be inferred from their findings, partly due to the small number of subjects. Their purpose was to shed light on which water use activities should be considered in the context of an epidemiologic study and to establish some practical approaches for future investigations. Noting the wide range in blood THM concentrations among the subjects in this and other studies in response to similar levels of THM exposure, subsequent exposure assessment research is being conducted on the possibility that genetic variation may play a role in individuals’ susceptibility to absorption of THMs.

## Figures and Tables

**Figure:**
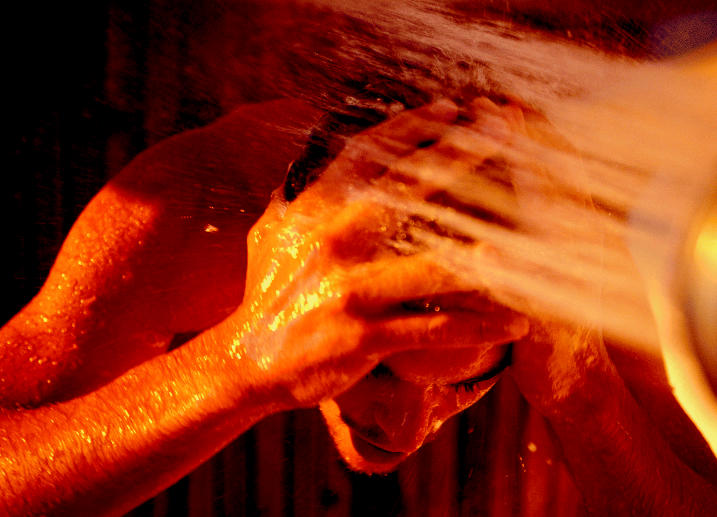
**Are shower buffs in hot water?** Household uses of hot tap water such as showering and dish washing result in greater THM absorption than simply drinking the water.

